# Hemolysis, Elevated Liver Enzymes and Low Platelet Count Syndrome Complicated by Disseminated Intravascular Coagulation and Posterior Reversible Encephalopathy Syndrome: A Case Report

**DOI:** 10.7759/cureus.59250

**Published:** 2024-04-29

**Authors:** Bhavana V Waghmare, Shubhada Jajoo, Dharmesh J Patel, Shazia Mohammad, Shaikh Muneeba

**Affiliations:** 1 Obstetrics and Gynaecology, Jawaharlal Nehru Medical College, Datta Meghe Institute of Higher Education and Research, Wardha, IND

**Keywords:** dysplastic intraperitoneal membrane, high-risk pregnancy, preeclampsia with hellp syndrome, posterior reversible encephalopathy syndrome (pres), disseminated intravascular coagulation (dic)

## Abstract

A 22-year-old pregnant woman was transferred from an external medical facility after experiencing an eclamptic seizure linked to hemolysis, elevated liver enzymes and low platelet count syndrome (HELLP) syndrome, and posterior reversible encephalopathy syndrome (PRES). Her situation was further complicated by intrauterine fetal demise and disseminated intravascular coagulation, necessitating a comprehensive multidisciplinary approach. This report details the diagnostic process and challenges in managing this complex patient with diverse medical requirements. Emphasis is placed on the observed hemostatic abnormalities, and we delineate the nuances in our approach compared to managing a similar condition in a nonpregnant patient. Heightened awareness among healthcare professionals is imperative for prompt diagnosis and effective intervention in such uncommon neurological complications during pregnancy.

## Introduction

Illustrating a case characterized by a clinical triad, we unveil the intricate interplay involving hemolysis, elevated liver enzymes, and low platelet count (HELLP) syndrome, disseminated intravascular coagulation (DIC), and posterior reversible encephalopathy syndrome (PRES). This unique clinical scenario emphasizes the complexities of obstetric complications and underscores the need for nuanced management to achieve favorable patient outcomes. The initial documentation of PRES traces back to 1996, credited to Hinchey et al. [1]. This condition has been recognized under various alternative terms, such as hypertensive encephalopathy, brain capillary leak syndrome, hyperperfusion encephalopathy, and reversible posterior leukoencephalopathy syndrome.

Weinstein delineated a distinct set of signs and symptoms, defining HELLP syndrome as a separate condition from severe preeclampsia [2]. While some categorize HELLP syndrome as a form of preeclampsia, it is also considered part of the spectrum of microangiopathic hemolytic anemias, alongside hemolytic uremic syndrome (HUS) and thrombotic thrombocytopenic purpura (TTP) [2,3]. HELLP syndrome reportedly affects 0.5-0.9% of pregnancies, impacting 10-20% of women with severe preeclampsia. Studies indicate a significant association between HELLP syndrome and DIC, ranging from 2.2% to 39% [4]. Managing patients diagnosed with PRES involves ensuring hemodynamic stability and hydration by administering intravenous crystalloids and colloids. Efforts are also directed toward maintaining optimal arterial oxygen pressure and addressing electrolyte disturbances and coagulopathy to enhance overall patient well-being. Timely diagnosis and treatment are crucial, as delayed identification may lead to enduring neurological complications [5,6]. Presented is a clinical case involving a 22-year-old pregnant woman diagnosed with HELLP syndrome, DIC, and PRES with fetal demise. This report delves into the diagnostic process, management strategies, fluid resuscitation approaches, and this patient's subsequent intensive care course, along with a comprehensive review of relevant medical literature [7].

## Case presentation

A 22-year-old primigravida at 35 weeks of gestation presented to the emergency department with complaints of convulsion episodes, loss of consciousness persisting for six hours, and frothing from the mouth over the same duration. She reported a history of amenorrhea for nine months and a previous diagnosis of hyperthyroidism treated with 50 mg of propylthiouracil once daily. The patient had no prior neurological or medical conditions. Upon examination, her blood pressure was 150/100 mmHg, her heart rate was 130 beats per minute, and her respiratory rate was 30 breaths per minute. The patient was found to be unconscious, with significantly reduced visual acuity. Neurological examination revealed brisk deep tendon reflexes and bilateral extensor plantar responses, indicative of central nervous system involvement. Her Glasgow Coma Scale (GCS) score was low, prompting intubation, with central and arterial lines inserted for monitoring.

Initial laboratory investigations, as detailed in Table 1, revealed significant findings. A 24-hour urine collection for protein showed marked proteinuria. There was a notable decrease in platelet count and haemoglobin levels, while liver enzyme, lactate dehydrogenase, C-reactive protein, and D-dimer levels were markedly elevated. Based on these laboratory findings and the patient's clinical symptoms, a diagnosis of HELLP syndrome, accompanied by DIC, was established.

**Table 1:** Laboratory parameters dL - Deciliter, g/dL - gram per deciliter, mg - milligram, U/L - unit per liter, mmol/L - millimoles per liter, ng/mL - nanograms per milliliter

Laboratory parameters	Patient’s values	Reference range
Hemoglobin	7.5 g/dL	13-17 g/dL
Total leukocyte count	10,000/dL	4000-11,000/dL
Platelet count	29,000/dL	150,000-400,000/dL
Serum creatinine	1.7 mg/dL	0.5-1.2 mg/dl
Serum urea	84 mg/dL	9-20 mg/dl
Serum potassium	3.3 mmol/L	3.5-5.1 mmol/L
Serum sodium	141 mmol/L	137-145 mmol/L
Serum magnesium	4.3 mg/dL	1.6–2.5 mg/dL
Albumin	3.4 g/dL	3.5-5.0 g/dL
Aspartate aminotransferase	3269 U/L	<50 U/L
Alanine aminotransferase	1687 U/L	17-59 U/L
Total bilirubin	1.8 mg/dL	0.2-1.3 mg/dL
LDH (Lactate dehydrogenase)	6138 U/L	140 to 280 U/L
D-dimer	7635 ng/mL	< OR = 500 ng/mL
C-Reactive protein (CRP)	53 mg/dL	< 3 mg/dL
Urine albumin	3+	-

**Figure 1:**
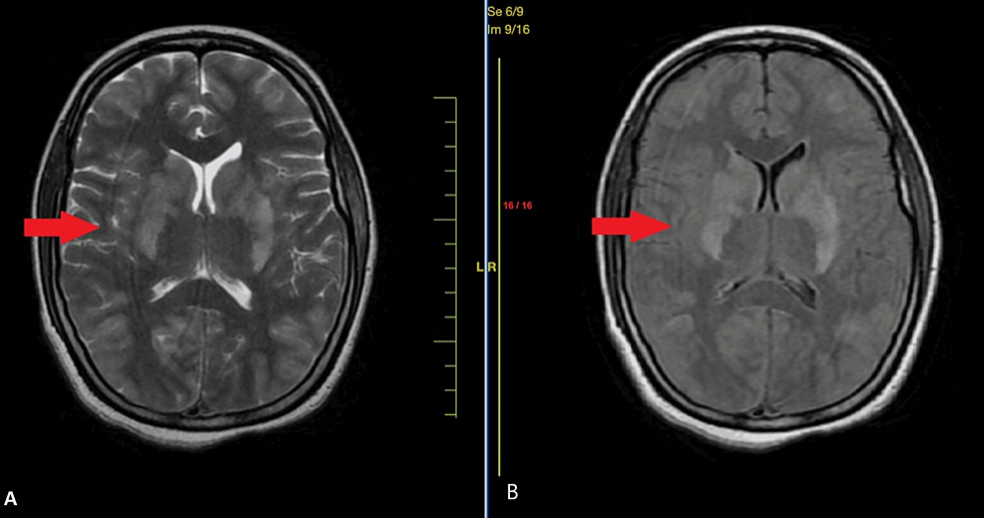
(A) High signal seen on the T2/FLAIR image in bilateral basal ganglia. (B) No restriction diffusion seen on DWI DWI: diffuse width imaging FLAIR: fluid-attenuated inversion recovery

The patient experienced contractions and delivered a male infant weighing 1.7 kg, who showed no signs of life and did not cry after birth, and suffered 2000 ml blood loss on delivery. During the patient's stay in the medical intensive care unit (MICU), she received 7 units of fresh frozen plasma, 8 units of random donor platelets, and 1 unit of whole blood to stabilize her condition. Brain magnetic resonance imaging (MRI), done upon consultation with a neurologist, revealed multiple regions of altered signal strength in the cortical and subcortical areas, affecting the temporal, high fronto-parietal, and bilateral perito-occipital lobes. Additionally, abnormalities were noted in the brainstem, perisylvian area, and bilateral basal ganglia, appearing hypointense on T1-weighted imaging but hyperintense on T2-weighted and fluid-attenuated inversion recovery (FLAIR) images. Diffuse width imaging (DWI) showed hyperintense signals in certain areas of the right occipital and front-parietal regions. These imaging findings suggest the presence of PRES (Figure 1).

The patient received prompt initiation of antihypertensive medication to regulate her blood pressure and was subsequently transferred to the neurology department for further assessment and treatment. A diagnosis of PRES associated with gestational hypertension, along with HELLP syndrome and DIC, was established. Treatment was initiated to lower the patient's blood pressure to safer levels. Additionally, she was administered intravenous medications, including a loading dose of levetiracetam (2 g), followed by maintenance doses of 1 g intravenously every 12 hours, and injection of lacosamide (400 mg) intravenously stat, followed by 200 mg intravenously every 12 hours for seizure prophylaxis. Injection mannitol (100 cc) was also administered intravenously. The patient's neurological symptoms gradually ameliorated with vigilant monitoring and supportive care over several days. As the patient's condition improved, her blood pressure stabilized, and her overall mental status showed enhancement, she was successfully weaned off the ventilator. After a hospital stay of 14 days, she underwent counselling sessions with neurologists and general physicians before being discharged. During the one-month follow-up, the patient was progressing well, and her visual field was noted to be normal.

## Discussion

PRES diagnosis relies on a clinical radiological approach, with validation from magnetic resonance (MR) brain scans, typical clinical features, and associated risk factors. Neurological manifestations may present singly or in combination and can progress throughout the acute phase. Classic symptoms include visual impairment, headaches, altered mental function, seizures, and nausea, with additional deficits such as weakness, sensory disturbances, or speech impairment. PRES has diverse origins, stemming from medical interventions like antineoplastic therapy or emerging as part of associated medical conditions such as autoimmune disorders or eclampsia [8,9]. PRES often presents with non-specific symptoms, gradually emerging over hours or days. Encephalopathy is observed in a significant percentage of cases, ranging from mild confusion to coma. Seizures affect a substantial proportion of patients, typically manifesting within 24 to 48 hours after presentation, with a minority progressing to status epilepticus [8,9]. The precise etiology of HELLP syndrome remains unclear, though it is commonly linked to issues such as endothelial dysfunction, immune maladjustment, and genetic predisposition. The sequence of events leading to DIC and PRES within the framework of HELLP syndrome is influenced by factors associated with pregnancy-induced hypertension.

HELLP syndrome is characterized by widespread endothelial injury, leading to hemolysis, elevated liver enzymes, and diminished platelet count. Damage to the endothelial layer and the release of procoagulant factors contribute to DIC, a systemic coagulopathy marked by both thrombotic events and bleeding tendencies. In the context of HELLP syndrome and DIC, compromised cerebral blood flow autoregulation and the potential for microvascular thrombosis increase the susceptibility to PRES. The pathophysiology of PRES involves vasogenic edema and alterations in blood-brain barrier permeability. Individuals presenting with HELLP syndrome may initially display nonspecific symptoms such as abdominal pain, nausea, and malaise. Neurological manifestations associated with PRES may include seizures, alterations in mental status, and visual disturbances. This intricate triad of symptoms can evolve rapidly, underscoring the importance of vigilant clinical observation [1].

DIC, a rare complication of pregnancy, has reported incidence rates ranging from 0.03% to 0.35%. It commonly occurs in conjunction with postpartum hemorrhage or placental abruption, although it can also be associated with conditions like HELLP syndrome or retained products of conception. Traditional clinical subtypes of DIC include balanced fibrinolysis (linked to malignancy), augmented fibrinolysis (associated with aortic aneurysms, hemorrhage, and placental abruption), and suppressed fibrinolysis (typical in sepsis) [10]. Identifying DIC in pregnant patients requires heightened awareness due to the unique laboratory changes observed during pregnancy. These alterations may include hyperfibrinogenemia (with third-trimester values typically around 5 g/L), decreased prothrombin time (PT), and partial thromboplastin time (aPTT), and thrombocytopenia. Therefore, deviations from non-pregnant patient norms in hematologic test results should prompt clinicians to exercise additional caution [11-13].

The management of DIC in obstetric patients prioritizes identifying and addressing the underlying cause, followed by transfusion of blood products and replenishment of coagulation factors, regular clinical and laboratory monitoring, and involvement of relevant specialists. While the infusion of blood products and clotting factors is crucial for managing acute hemorrhage, careful consideration must be given to prevent the potential risk of inducing acute thrombosis. In cases of DIC complicated by thrombosis, therapeutic heparin administration may be considered. However, this approach is contraindicated in the presence of active hemorrhage or in patients with a heightened risk of bleeding, such as parturients [14,15]. Individualized management is essential for HELLP syndrome complicated by DIC and PRES, taking into account the severity of symptoms, gestational age, and overall clinical condition of the patient. Regular reassessment and collaboration among various medical specialties are vital to achieve optimal outcomes for both mother and infant.

## Conclusions

In conclusion, this case report elucidating the rare incidence of HELLP syndrome, complicated by DIC and PRES, serves as a notable example of intricate obstetric complications necessitating comprehensive management. A thorough examination of this case will aid obstetricians and the medical community in grasping the complexity of clinical presentation and treatment modalities in similar cases. While there remains considerable opportunity to formulate guidelines to address such occurrences, this case report constitutes a valuable addition to the comprehension of and approach toward complex obstetric situations involving HELLP syndrome, DIC, and PRES.
